# Supplementary material for: Resonantly Pumped Bright-Triplet Exciton Lasing in Cesium Lead Bromide Perovskites
Source: ACS Photonics. 2021 Aug 27;8(9):2699–704. doi: 10.1021/acsphotonics.1c00720 (PMC8451394; doi:10.1021/acsphotonics.1c00720)
Supplement: Supplementary file 1 — ph1c00720_si_001.pdf [file ph1c00720_si_001.pdf]

Supporting Information

Resonantly pumped bright-triplet exciton lasing in caesium lead bromide perovskites

Guanhua Ying* [1], Tristan Farrow* [1,2,†], Atanu Jana[3], Hanbo Shao[4], Hyunsik Im[3], Vitaly Osokin[1], Seung Bin Baek[5], Mutibah Alanazi[1], Sanjit Karmakar[2], Manas Mukherjee[2,†], Youngsin Park[5,†], Robert A. Taylor[1,†]

* Co-first authors

† Corresponding Authors, Tristan Farrow (Tristan.Farrow@cantab.net), Manas Mukherjee (cqtmukhe@nus.edu.sg), Youngsin Park (ysinpark@unist.ac.kr), Robert Taylor (robert.taylor@physics.ox.ac.uk)

[1]Clarendon Laboratory, Department of Physics, University of Oxford, Parks Road, Oxford OX1 3PU, UK

[2]Centre for Quantum Technologies, National University of Singapore, Science Drive 2, Singapore 117543

[3]Division of Physics and Semiconductor, Dongguk University, Seoul 04620, Korea. [4]State Key Laboratory of Mechanics and Control of Mechanical Structures, Nanjing University of Aeronautics and Astronautics, China

[5]School of Natural Science, Ulsan National Institute of Science and Technology, Ulsan 44919, Korea

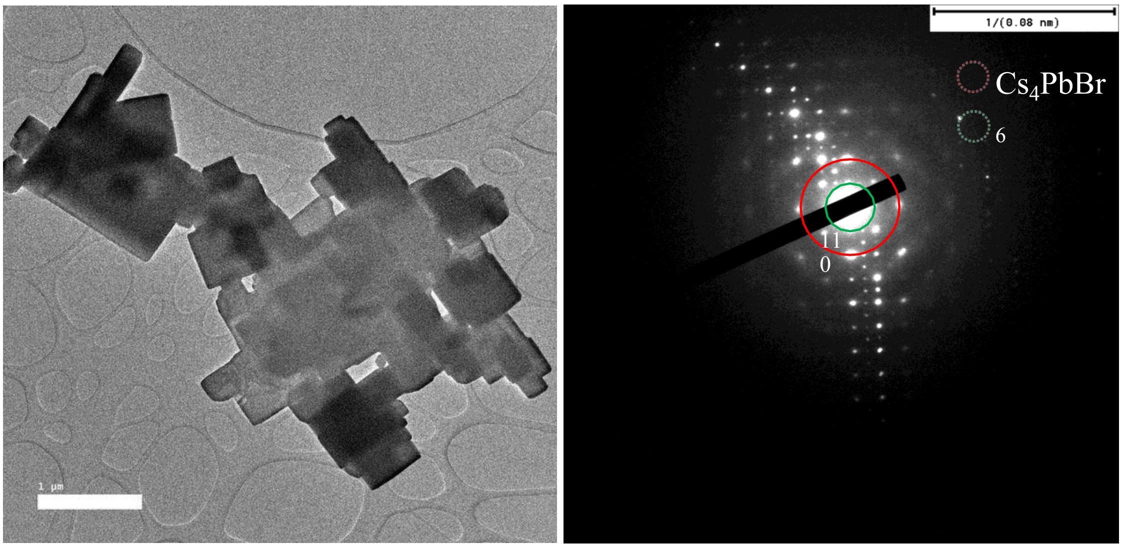

Fig. S1| TEM image (left) of $CsPbBr_3$ nanocrystals with cuboid symmetry. The thickness of the sample does not allow fringes of the lattice to be visible but the Selected Area Electron Diffraction (SAED) pattern (right) of the sample corroborates the presence of $CsPbBr_3$ nanocrystal islands encapsulated in Cs_4PbBr_6 microcrystals with rhombic symmetry.

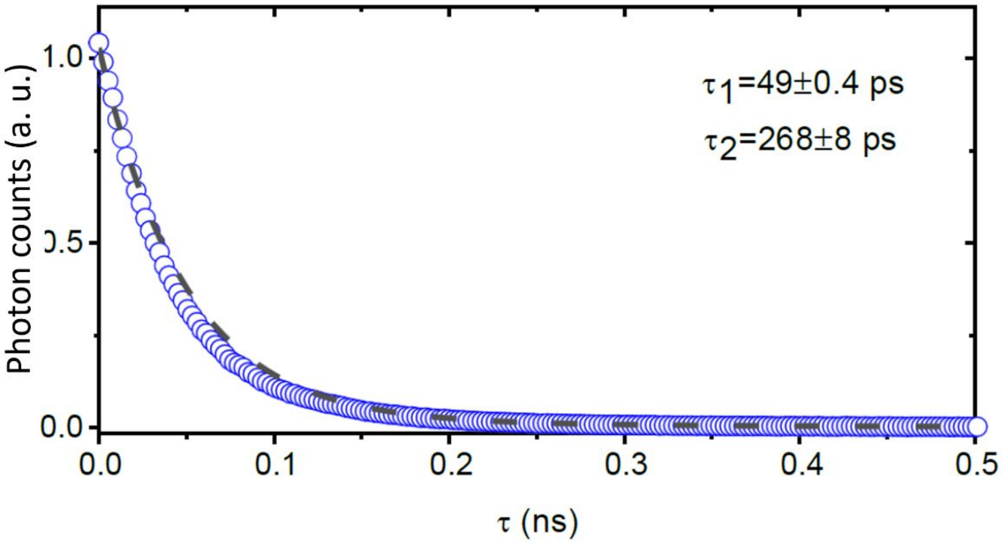

Fig. S2 | Time-resolved PL decay of $CsPbBr_3$ nanocrystal pumped quasi-resonantly.

The decay fit was produced using *PicoQuant EasyTau* software to extract the time constants for the fast signal (49 ps) due to stimulated emission and the delayed contribution (268 ps) from spontaneous emission convolved with the instrument response function. The spontaneous lifetime corroborates a weak confinement regime in nanocrystals where the Bohr radius of the exciton is ~10 nm.

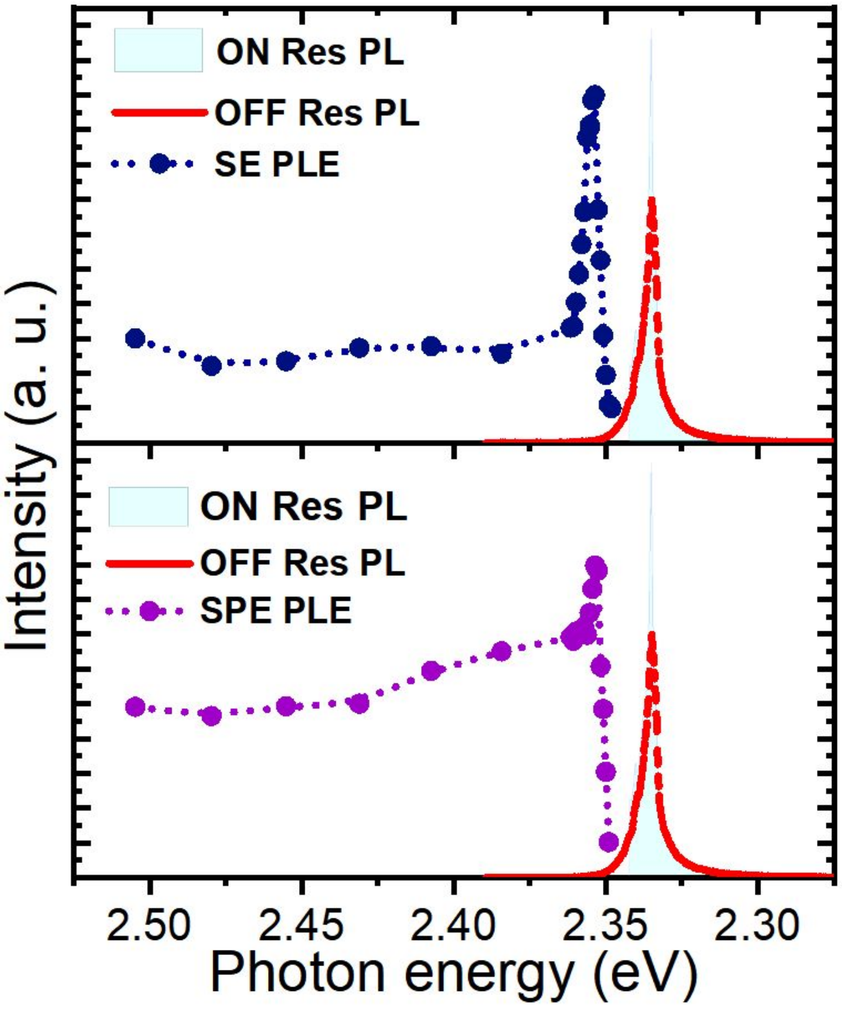

Fig. S3 | PLE and PL plot of on – and off resonant.

PLE plot for SE (dark blue dotted line) and SPE (violet dotted line) signals respectively. The red profile shows the PL emission from non-resonant pumping and the light blue shadow indicates that obtained from near-resonant excitation. The SE PLE peak is Stoke-shifted by 18 meV with respect to the SE PL peak, and suggests small losses due to vibrational relaxation.

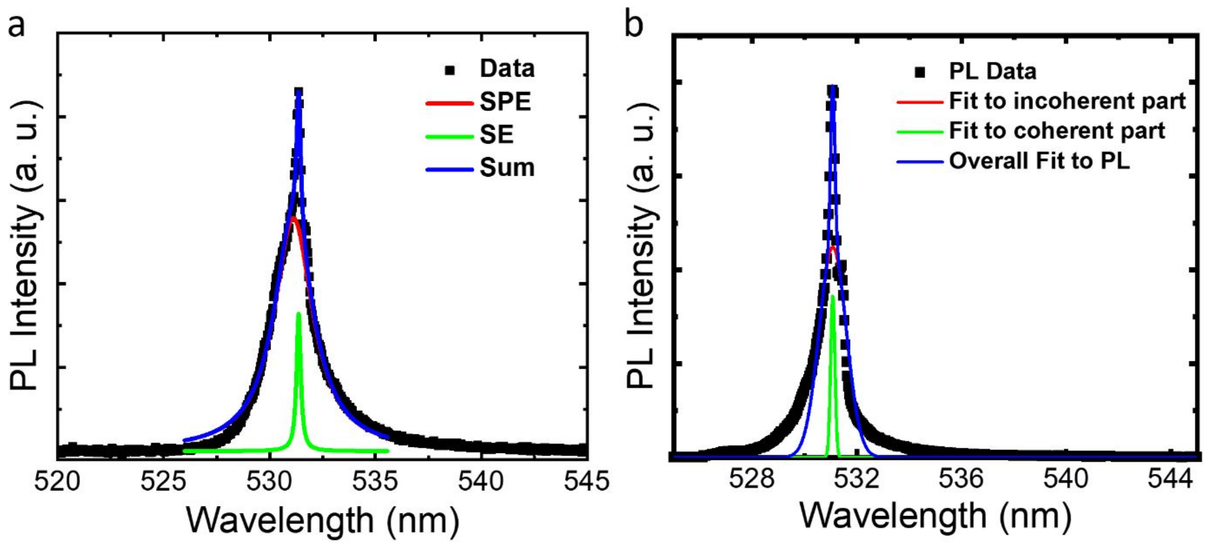

Fig. S4| PL fitting.
(A) Fitting of off-resonant PL emission using Gaussian function measured at 25 mW pump power. **(B)** Fitting of the coherent and incoherent components of the $CsPbBr_3$ nanocrystal emission signal from the time integrated PL spectrum.

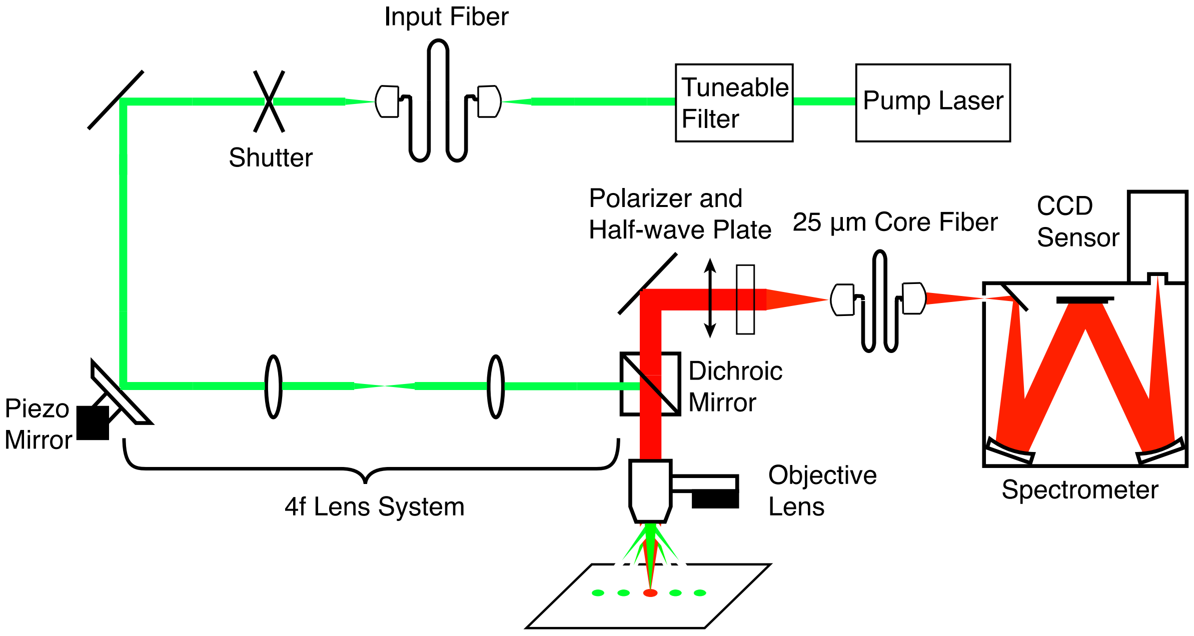

Fig. S5| Diagram of apparatus.
Diagram of the telecentric optical system used to excite the perovskite microcrystals and the confocal collection system used to gather the luminescence. The objective lens is mounted on a piezoelectric xyz and can be scanned independently of the excitation. Polarizer and half wave plate were used together to analyze the emission and remove any influence from the polarization dependence of the spectrograph and CCD.
